# Impact of calcium on N1 influenza neuraminidase dynamics and binding free energy

**DOI:** 10.1002/prot.22761

**Published:** 2010-03-05

**Authors:** Morgan Lawrenz, Jeff Wereszczynski, Rommie Amaro, Ross Walker, Adrian Roitberg, J Andrew McCammon

**Affiliations:** 1 Department of Chemistry and Biochemistry, University of California,San Diego, La Jolla, California; 2 Center for Theoretical Biological Physics, University of California,San Diego, La Jolla, California; 3 Department of Pharmaceutical Sciences, University of California,Irvine, California; 4 Department of Computer Science, University of California,Irvine, California; 5 San Diego Supercomputer Center, University of California,San Diego, California; 6 Quantum Theory Project, University of Florida,Gainesville, Florida; 7 Department of Pharmacology, UCSD,La Jolla, California; 8 Howard Hughes Medical Institute, University of California,San Diego, La Jolla, California

**Keywords:** oseltamivir, thermodynamic integration, Bennett Acceptance Ratio, force field comparison, metal binding, molecular dynamics

## Abstract

The highly pathogenic influenza strains H5N1 and H1N1 are currently treated with inhibitors of the viral surface protein neuraminidase (N1). Crystal structures of N1 indicate a conserved, high affinity calcium binding site located near the active site. The specific role of this calcium in the enzyme mechanism is unknown, though it has been shown to be important for enzymatic activity and thermostability. We report molecular dynamics (MD) simulations of calcium-bound and calcium-free N1 complexes with the inhibitor oseltamivir (marketed as the drug Tamiflu), independently using both the AMBER FF99SB and GROMOS96 force fields, to give structural insight into calcium stabilization of key framework residues. Y347, which demonstrates similar sampling patterns in the simulations of both force fields, is implicated as an important N1 residue that can “clamp” the ligand into a favorable binding pose. Free energy perturbation and thermodynamic integration calculations, using two different force fields, support the importance of Y347 and indicate a +3 to +5 kcal/mol change in the binding free energy of oseltamivir in the absence of calcium. With the important role of structure-based drug design for neuraminidase inhibitors and the growing literature on emerging strains and subtypes, inclusion of this calcium for active site stability is particularly crucial for computational efforts such as homology modeling, virtual screening, and free energy methods. Proteins 2010. © 2010 Wiley-Liss, Inc.

## INTRODUCTION

Type A influenza virus is becoming a worldwide pandemic threat due to its virulence and transmissibility in people.* The viral surface protein neuraminidase, along with hemagglutinin, classifies influenza subtypes and fulfills an important role in viral propagation by cleaving a terminal sialic acid from host cell surfaces; thus, neuraminidase is a key drug target for controlling flu infection. The enzyme has two distinct families,^1^ group 1 (N1, N4, N5 and N8) and group 2 (N2, N3, N6 and N7), with group 1 including the N1 subtype found in recent outbreaks of the highly pathogenic subtype H5N1, as well as the 1918 epidemic and “swine flu” H1N1. The protein exhibits a high rate of antigenic shift and drift, yielding proteins with heterogeneous sequences that can reduce sensitivity to the clinically used inhibitors oseltamivir (marketed as Tamiflu by Roche Pharmaceuticals, Basel, Switzerland), zanamivir (marketed as Relenza by GlaxoSmithKline, Bentford), and peramivir (BioCryst Pharmaceuticals, Birmingham, AL).

A high-affinity calcium binding site near the active site has been identified through crystallography1–4 and Proton Induced X-ray Emission (PIXE) experiments,^5^ though the calcium density was not resolved or discussed for some holo structures of group 1 neuraminidase.^1^ The specific role of this calcium ion is unknown, but experiments have supported its importance for wild-type enzyme activity^6^ and enzyme thermostability^7^. Commonly performed fluorometric neuraminidase activity assays include calcium salts in the activity buffer and follow the protocol developed originally by Potier *et al.*,^8^ where increased calcium ion concentration was found to augment activity. In addition, a recent crystal structure of calcium-deficient group 2 neuraminidase shows destabilization of the key active site residue R292 near the calcium binding site.^9^

Although dynamics of N1 ligand binding have been examined computationally,10–13 no dynamic, structural study has investigated the contribution of this calcium to neuraminidase substrate binding. Here, we compare simulations of calcium-bound and calcium-free complexes of N1 neuraminidase bound to the FDA-approved, nanomolar-affinity drug oseltamivir. To quantify the impact of this calcium on oseltamivir binding affinity, we calculate the free energy of binding for both complexes. This study also checks for force field dependency of the observed calcium effects on N1 dynamics through comparison of simulations and free energy calculations performed with the AMBER FF99SB and GROMOS96 force fields.

We emphasize the impact of calcium binding on integrity of the active site and accurate calculation of binding free energies. In particular, for N1, calcium stabilizes Y347 for interaction with the ligand and helps to maintain an optimal binding pose with other N1 binding site residues. Incorporation of calcium as an integral part of the active site is particularly crucial given the importance of structure-based drug design for N1 inhibitor development to date^14^ and prevalance of homology models^15^, ^16^ for emerging subtypes and resistance mutations, virtual screening,^17^, ^18^ and free-energy methods19–21 for this protein.

## MATERIALS AND METHODS

N1 monomer simulations were performed using the GROMOS05 software for biomolecular simulation^22^ and the GROMOS96 force field (45A3 parameter set).^23^ Molecular Dynamics (MD) set up is described in detail in the Supporting Information Methods section. Both complexes of N1-oseltamivir, with and without the bound calcium ion, were simulated in 10 independent trajectories, each 4 ns, for a total of 40 ns of simulation for each complex. Comprising the 10 simulations were five simulations generated from the chain B monomer in the oseltamivir-bound Loop 150 “open” crystal structure (PDBID: 2HU0) and five simulations started from the chain A monomer in the holo Loop 150 “closed” crystal structure (PDBID: 2HU4). As the calcium density was not present in these crystal structures, overlap with the apo N1 structure 2HTY aided in positioning of the ion in the protein; the calcium was parametrized in the classical force field. To generate the independent trajectories, these structures were each initialized with random velocities assigned from a Maxwell–Boltzmann distribution at 5 K.

Two 100 ns N1 tetramer simulations with the AMBER FF99SB force field^24^, ^25^ were performed, each with atomic coordinates taken from the holo, open Loop 150 crystal structure (2HU0) and with the calcium inserted from overlap with the apo 2HTY structure. Calcium-bound simulations used the PMEMD module in AMBER 10,^26^ whereas calcium-free simulations were performed using the Desmond Molecular Dynamics package developed by D.E.Shaw Research.^27^ For details of these simulations, please see the Supporting Information Methods section. Although there are a few differences in the specifics of the MD engines used to run the AMBER FF99SB simulations (Supporting Information Methods), the similarity in RMSF (Supporting Information Fig. 1) indicates similar sampling of conformational space. AMBER FF99SB trajectories for each monomer of the tetramer were extracted and concatenated to approximate 400 ns of monomer N1 sampling.

Analysis tools in Visual Molecular Dynamics (VMD)^28^ were applied for calculation of root mean squared fluctuation (RMSF) and deviation (RMSD), and monitoring of torsion, hydrogen bonds, and salt bridge distances. Hydrogen bond criteria applied a donor–acceptor distance cutoff of 3.5 Å and a 120° cutoff for the donor-hydrogen-acceptor angle. For analysis, structures were extracted every 10 ps for each 400 ns AMBER FF99SB trajectory and 2 ps for the 40 ns GROMOS96 trajectories. These structures were used for the GROMOS RMSD clustering algorithm,^29^ applied using the cluster2 program in GROMOS05 software.^22^ A rotational and translational fitting was applied to all the C_α_ carbons, followed by application of the RMSD clustering to all atoms of 41 residues that comprise the binding site (residues 117–119, 133–138, 156, 178–179, 196–200, 223–228, 243–247, 276, 277, 292–295, 344–347, 371, 401, 402). This selection is similar to that used for relaxed complex docking of N1 in Cheng *et al*.,^18^ but here we exclude residues in the highly flexible Loop 150 (residues 147–152) and Loop 430 (430–439) regions to focus the algorithm on the active site portion near the calcium binding site. After screening a variety of RMSD cutoff values for cluster generation, using the total number and diversity of clusters as criteria, a value of 1.5 Å was selected for the GROMOS96 trajectories, and a value of 1.2 Å for AMBER FF99SB trajectories. The discrepancy in the chosen RMSD cutoff, as well as the varied cluster populations, can be attributed to the varied potential energy landscapes produced by the different force fields, as well as the use of multiple, shorter GROMOS96 trajectories and fewer, longer AMBER FF99SB simulations.

Two different, rigorous binding free energy calculations were used: free energy perturbation (FEP), with the AMBER FF99SB force field, and thermodynamic integration (TI) with the GROMOS96 force field. Both bound and unbound calculations were performed, to give a Δ*G*_protein_ from decoupling the bound oseltamivir from N1, and a Δ*G*_water_ from decoupling of unbound oseltamivir, free in a box of water. Following the thermodynamic cycle involved in double decoupling,^30^, ^31^ Δ*G*_protein_ is subtracted from Δ*G*_water_ to give Δ*G*_bind_.

The GROMOS05^22^ software was used for both oseltamivir-bound and oseltamivir-unbound TI calculations, repeated in independent trajectories (IT-TI). For the bound state decoupling, six calculations decoupled oseltamivir from the ion-bound complex, and six decoupled the ligand from the ion-free complex, for a total of 12 N1 TI calculations. For each of these complexes, the calculations were performed in triplicate, with different starting structures that capture diverse conformations of Loop 150; three calculations were initialized from a closed Loop 150 N1 conformation, and three were initialized from an open Loop 150 conformation. These starting structures were extracted from the independent MD trajectories described above, after 1 ns of equilibration. Six independent oseltamivir-unbound TI calculations decoupled oseltamivir from a 37 × 37 × 37 Å^3^ box of SPC water. For each TI, 26 windows span the λ range from 0.00 (ligand coupled) to 1.00 (ligand decoupled), in 0.04 intervals. For ligand–protein decoupling, a 500 ps equilibration period preceded a 500 ps sampling period, from which 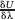 data was recorded; the unbound decoupling calculations involved 400 ps for both equilibration and sampling periods per λ window.

To maintain a defined bound state in the protein calculations, a 0.6 kcal/mol/Å^2^ harmonic restraint constant was applied to the C4 atom of oseltamivir and the associated volume correction^31^ + *RT*ln(*CV*) for the free energy was calculated to be −5.5 kcal/mol. Soft-core interaction potentials^32^ (*s*_LJ_ = 0.5 and *s*_C_ = 0.5) were used for ligand atoms involved in the perturbation to avoid end point singularities and to enhance sampling. For each λ window, the statistical inefficiency of the raw 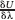 data was calculated using the method described and coded by Chodera *et al*.^33^ The decorrelated data was averaged to approximate 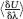, which was integrated by the trapezoidal rule over the 26 λ windows for each of *N*= 6 independent calculations, as:


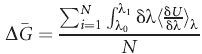
(1)

Subtraction of the Δ*G*_protein_ from Δ*G*_water_ gives a Δ*G*_bind_. A standard error 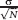 was computed for both Δ*G*_protein_ and Δ*G*_water_ over the *N* = 6 independent runs and propagated for Δ*G*_bind_ (listed in Table III).

To examine the advantage of shorter, independent trajectories over a single, long trajectory in the TI calculations, one run for each set of protein TI calculations was selected for the extension of equilibration and sampling times (1 ns equilibration and 3.8 ns sampling per λ), for an 85.8 ns increase in total sampling time for the calculation. The single Δ*G*_protein_ produced from each extended run differed from that of the “short” calculation result by <4%; however, the associated Δ*G*_bind_ was +3.3 kcal/mol less favorable than the experimentally derived target value. This discrepancy is larger than that for the IT-TI result for Δ*G*_bind_, which is off the target value by +1.3 kcal/mol (Table III). The extended ion-free result was also significantly more unfavorable than the IT-TI value. This indicates that these single, extended simulations were unable to overcome sampling barriers to generate a complete ensemble average 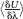. Combining multiple TI calculations initialized from diverse conformations improves sampling and can result in a more correct Δ*G*_bind_.^12^, ^34^, ^35^

Free energy calculations on the tetrameric complex were performed with the AMBER FF99SB force field in Desmond using FEP. For both the decoupling of unbound oseltamivir (to calculate Δ*G*_water_) and bound oseltamivir (for Δ*G*_protein_), 21 windows were used, one with full non-bonded interaction, 10 for the annihilation of columbic interactions (with full van der Waals interactions), and 10 with the removal of van der Waals interactions (with no electrostatics). A softcore potential was used with α = 0.5. Decoupling of unbound oseltamivir in water was performed on a system of the ligand in a 24 × 28 × 29 Å^3^ TIP3P water box with 250 ps of equilibration followed by 1 ns of sampling in each window. Bound oseltamivir decoupling calculations were performed on the tetrameric complex (from 2HU0, described above) with all four ligand molecules simultaneously removed to enhance sampling. Following 5 ns of equilibration of the starting structure, each λ window was individually equilibrated for 1 ns and then sampled for 2 ns.

Three sets of protein–ligand calculations were performed, two with the protein coordinates from the 2HU0 crystal structure (one with the bound calcium and one without) and another without calcium and initiated from clustering results in which Y347 is flipped out of the pocket (see below). Reported work functions were post-processed with the Multistate Bennett Acceptance Ratio (MBAR).^36^ Energies were decorrelated based on their statistical inefficiencies, as in the GROMOS96 TI simulations, and error bars representing the analytic errors developed in the MBAR implementation, here represented by σ, were calculated with code made publicly available by Shirts and Chodera.† The free energy of decoupling the four ligands from the N1 tetramer was divided by four to obtain an average binding free energy to the N1 monomer, with a standard error 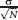 calculated using *N* = 4.

## RESULTS AND DISCUSSION

### RMSF

**Figure 1:**
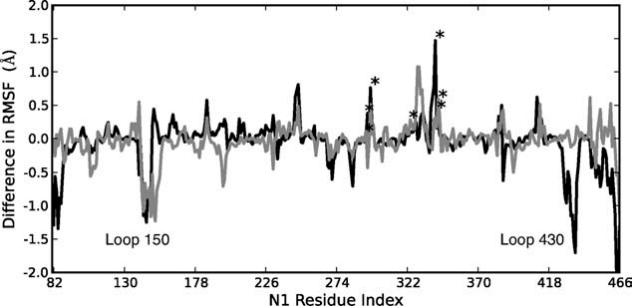
Cα RMSF differences for ion-free and ion-bound simulations. GROMOS96 (gray) and AMBER FF99SB (black) ion-bound RMSF is subtracted from the ion-free simulation RMSF. Asterisks indicate residues that contact the calcium ion in the 2HU0 structure.

The structure of the N1 calcium binding site includes close ion contacts with the backbone carbonyls of D293, N294, G297, G345, A346, Y347, and the carboxyl groups of both D293 and D324, with similar interactions observed in group 1 and 2 crystal structures.1–4, 9 Therefore, removal of the ion can be expected to destabilize these residues and the loops on which they are located, leading to increased fluctuation. Both GROMOS96 and AMBER FF99SB ion-bound MD simulations were checked for similar RMSF per residue C_α_ (Supporting Information Fig. 1), with the most flexibility observed for Loop 150, Loop 430, and portions of a long disordered loop (residues 327–348) surrounding the calcium binding site. Plots of the difference in ion-free and ion-bound simulation RMSF (Fig. .1) reveal changes in backbone RMSF for these regions. Both AMBER FF99SB and GROMOS96 simulations indicate increased fluctuation near the calcium-contacting residues (see peaks near asterisks in Fig. .1), with some of the peaks corresponding to residues very close to the active site. RMSF changes are color-mapped onto the N1 monomer structure in Supporting Information Figure 2, for an overall view of where these changes occur in the protein. Loop 150 and 430 (labeled in Fig. .1) have changes in flexibility that are inconsistent in comparison of the GROMOS96 and AMBER FF99SB simulations.

### Y347 torsion

**Figure 2:**
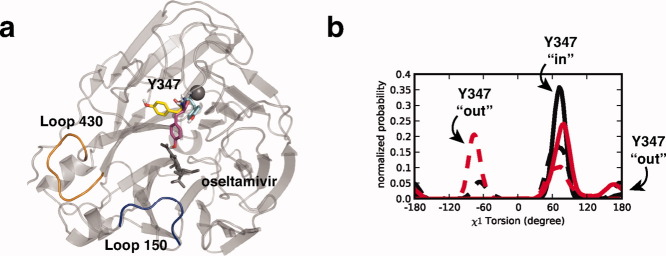
Y347 Sampling in the N1 Active Site. (a) The N1 monomer in complex with oseltamivir (gray) and with bound calcium (gray), as well as the Y347 “in” conformation (pink) and two representative “out” conformations (yellow and cyan). (b) Normalized probability distributions for the Y347 χ1 sidechain torsion plotted for ion-bound AMBER FF99SB (black, solid lines) and GROMOS96 (red, solid lines) simulations, compared to the corresponding distributions for ion-free simulations (dashed lines).

Of all residues with increased mobility in the N1 ion-free simulations, Y347 has the most direct impact on ligand binding. The Y347 backbone carbonyl coordinates calcium in the N1 crystal structures^1^, ^4^ [pink conformation in Fig. .2(a)], stabilizing the residue for interaction with the conserved ligand carboxyl group. In Figure 2(b), distributions for the tyrosine χ1 torsion indicate a change of Y347 conformation in the ion-free simulations (dashed vs. solid lines). Torsions within the range 30–105° designate conformations that direct the sidechain into the active site for hydrogen bonding to the ligand (“in” conformation), whereas other torsion ranges correspond to conformations flipped out of the pocket and inaccessible to the ligand (“out” conformation). With the loss of its stabilizing backbone contact with calcium, Y347 increases sampling of “out” conformations [see cyan and yellow conformations in Fig. .2(a)].

Integration of the curves in Figure 2(b) allows for the estimation of the total time spent in the “out” Y347 conformations, whereas tracking the torsion time series indicates how often transitions occur (Supporting Information Fig. 3). In the ion-free simulations, the destabilized Y347 flips out of the pocket for 54% of the GROMOS96 simulation, and for 29% of the AMBER FF99SB simulation. Examination of each 100-ns monomer AMBER FF99SB trajectory reveals an initial “flip” after 5 ns in the chain B simulation, but no flip is seen before 13, 28, and 58 ns for chains A, C, and D, respectively (see Supporting Information Fig. 3). Five of the 10, 4 ns GROMOS96 ion-free simulations were initialized in a Y347 “out” conformation, four of which do not visit the “in” conformation. Those initialized with the “in” Y347 conformation flipped within 1.4 ns, whereas one run remained unflipped throughout 4 ns (Supporting Information Fig. 3). Thus, although the varied AMBER FF99SB and GROMOS96 simulation lengths prohibit a direct comparison of duration for the Y347 flipped conformation, the GROMOS96 simulations seem to allow more facile Y347 flipping than do the AMBER FF99SB simulations.

The “in” ligand-accessible conformation is dominant in the ion-bound simulations, with the “out” conformation occurring for only 20% and 4% of the GROMOS96 and AMBER FF99SB simulations, respectively. We note that not all conformations in the “in” torsion range result in hydrogen bond formation, but the Y347 phenol is directed toward the ligand carboxyl for potential interaction.

### Hydrogen bonds

The increased sampling of Y347 “out” conformations in the ion-free simulations reduces the availability of this residue for hydrogen bonding to oseltamivir. Hydrogen bond analysis using geometric criteria (see Methods) reveals an overall reduction in the occurrence of hydrogen bonds formed between the protein and ligand for the ion-free simulations (Table I). Y347 hydrogen bond occurrence is reduced by 27% in the GROMOS96 ion-free simulations and by 14% in the AMBER FF99SB ion-free simulations.

**Table I:** Change in % Occurrence of N1-Oseltamivir Hydrogen Bonds in Ion-Free Simulations Relative to Ion-Bound Simulations

Ligand moiety	Carboxyl					Ammonium								Acetamide									
	R118	R292	R371	Y347	E119	E227	E277	E227	E277
AMBER FF99SB GROMOS96	+6	−13	−13	−14	−30	None^a^	None	None	None
	−8	−40	−21	−27	−18	−49	−35	−16	−3

a Indicates no hydrogen bond observed to meet criteria described in Methods.

Calcium removal affects the stability of other key binding site residues as well, resulting in reduced occurrence of hydrogen bonds to the ligand. The arginine triad, an important neuraminidase binding motif that includes R118, R292, and R371, interacts with the carboxyl of ligands.^1^ A large loss of ligand hydrogen bonds to R292 and R371 is observed in the ion-free GROMOS96 simulations, and less severe but significant reductions are also observed for these residues in the AMBER FF99SB ion-free simulations. In the AMBER FF99SB simulations of both complexes, R118 only weakly meets hydrogen bonding criteria for the ligand.

With further comparison of changes in hydrogen bond occurrence, a difference in sampling becomes evident for the simulations with two different force fields. A significant reduction in hydrogen bond occurrence between the oseltamivir ammonium and the E119 carboxyl group is observed for both force fields, but the GROMOS96 simulations favor interactions of the ligand ammonium with E227 and E277; in the AMBER FF99SB simulations this ligand moiety is consistently oriented toward E119. Hydrogen bonds to residues on the flexible Loop 150 are also sampled differently in the AMBER FF99SB and GROMOS96 simulations (Supporting Information Table I). This inconsistency is also seen in the RMSF changes in Figure 1, and could be due to the different MD protocols used for GROMOS96 and AMBER FF99SB simulations, as each monomer of the AMBER FF99SB simulation was initialized in the same open Loop 150 conformation, but allowed to run for much longer periods compared to the GROMOS96 simulations (see Methods). In the AMBER FF99SB ion-free simulation, ligand hydrogen bonds to Loop 150 residues D151 and R152 are significantly reduced (Supporting Information Table I) and Loop 150 is observed to sample primarily open states (Supporting Information Fig. 4).

The protein–ligand interactions described in Table I are also largely electrostatic; monitoring changes in the distance between the center of mass for these reacting moieties [Supporting Information Fig. 5(a–d)] confirm the breaking of salt bridges and corroborate the hydrogen bonding data.

### Clustering and structural changes

RMSD clustering reduces the simulation structural data to a set of representative conformations for analysis. Clusters that together capture at least 85% of the total simulation ensemble are listed in Table II, with the conformation of Y347 indicated. The varied cluster populations in Table II also highlight differences in the AMBER FF99SB and GROMOS96 simulations. The cutoffs were chosen to balance resolution of unique clusters with reduction of the simulation data to a manageable number of representative conformations. The higher selected cutoff for the GROMOS96 simulations as well as the increased total number of clusters show that both GROMOS96 simulations allow more fluctuations and increased heterogeneity in sampling of conformational space; this was also indicated by higher dimensionality for GROMOS96 essential dynamics found in a broad force field comparison study.^37^ The difference in sampling could also be attributed to the fact that the GROMOS96 simulations, which consist of ten 4 ns trajectories, may benefit from increased sampling due to the use of multiple, shorter trajectories,^38^, ^39^ as opposed to the AMBER FF99SB simulations, which consist of fewer (four), longer (100 ns) trajectories.

**Table II:** Summary of Cluster Populations

	GROMOS96					AMBER FF99SB								
Ion			No Ion					Ion							No Ion								
Cluster^a^	% Ensemble	Y347	% Ensemble	Y347	% Ensemble	Y347	% Ensemble	Y347
1	24	in	39	out	93	in	50	in
2	12	in	11	in			18	out
3	9	in	10	in			15	out
4	8	out	9	out			13	out
5	8	in	9	in				
6	8	in	9	out				
7	8	out	8	out				
8	7	out						

a Clusters listed combine to represent at least 85% of the total ensemble of configurations from the simulation.

**Figure 3:**
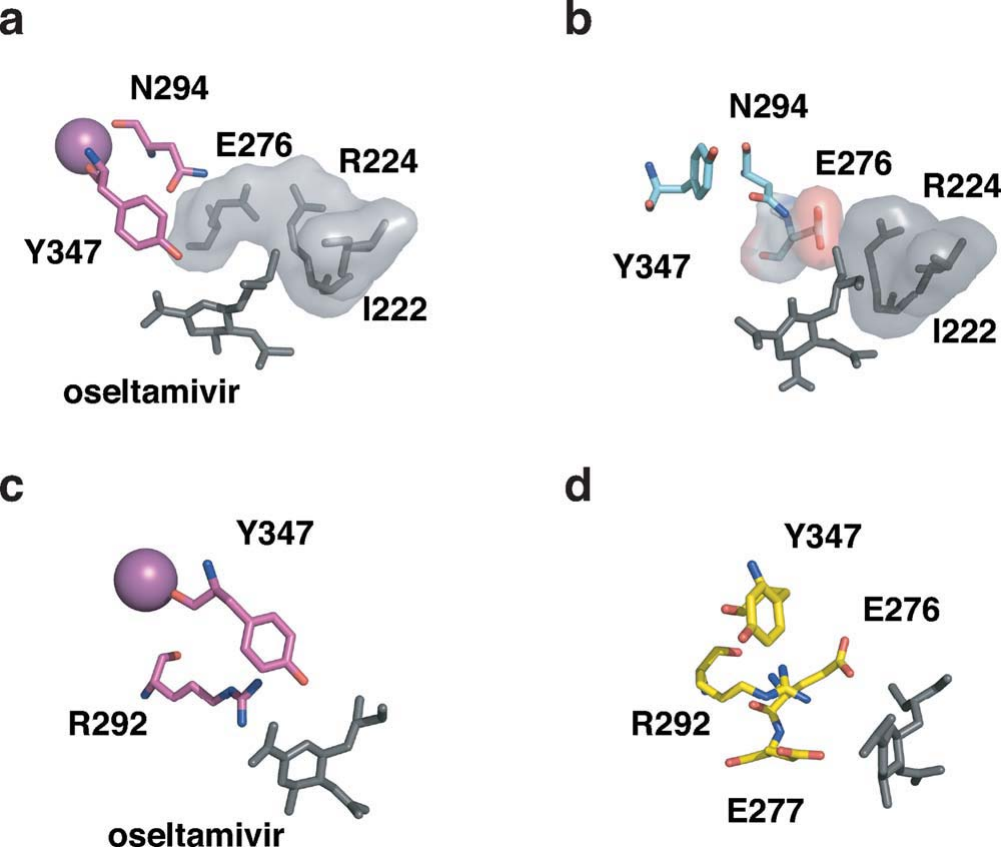
Binding pose comparison for ion-bound and ion-free simulations. Oseltamivir is shown in dark gray for all panels, with residues from the top cluster of the GROMOS96 ion-bound simulation (pink) in panels (a) and (c), from the cluster four of the GROMOS96 ion-free simulation (yellow) in panel (b), and from the cluster two of the AMBER FF99SB ion-free simulation (cyan) in panel (d). The disturbed hydrophobic subpocket structure is shown with surface representation in (b) and the retreated R292 conformation in (d).

Structural differences observed by comparison of cluster central members for the different force fields largely correspond to sampling differences observed in hydrogen bonding data (Table I) and RMSF changes (Fig. .1). However, both AMBER FF99SB and GROMOS96 ion-free clusters indicate changes in position of Y347 and N294, which both have backbone carbonyls directed toward the calcium and have sidechains that extend into the active site [Fig. .3(a,c)]. Other common differences occur on a solvent exposed loop near the rim of the binding site, in the 247–249 region (Fig. .1 and Supporting Information Fig. 2). Figure 3 has examples of conformational changes represented in the ion-free GROMOS96 and AMBER FF99SB clusters, which could be most pertinent for accuracy of docking or other computational free energy methods.

Destabilization of N294 due to its lost backbone contact to the calcium enables the N294 polar sidechain to shift closer into the hydrophobic subpocket^40^ that accepts the oseltamivir aliphatic tail [Fig. .3(a,b)]. This shift is further revealed by tracking the proximity of the N294 sidechain to the oseltamivir aliphatic tail throughout all simulations [Supporting Information Fig. 5(e,f)]. Instability of this residue, as well as Y347, has been proposed as the source of resistance to oseltamivir conferred by the N294S mutation in N1.^4^ In the GROMOS96 simulations, changes in R292 and E276 conformations also bring these highly polar residues closer to the hydrohobic tail of oseltamivir [Supporting Information Fig. 5(e)].

Another significant conformational change is observed in cluster four for the GROMOS96 ion-free simulation. Here, Y347 is flipped out of the pocket, and the nearby residue R292 retreats away from its electrostatic interaction with the oseltamivir carboxyl to interact with flanking glutamates E276 and E277 [Fig. .3(c,d)]. This perturbed R292 conformation is also observed in a calcium deficient crystal structure of N9 neuraminidase.^9^ In the AMBER FF99SB ion-free simulations, interaction with oseltamivir and R292 is weakened (Table I), but this conformational change is not observed.

### Binding free energies

To quantify the impact of the aforementioned changes in dynamics of the N1-oseltamivir complex in absence of calcium, parallel free energy calculations were performed: free energy perturbation (FEP) with the AMBER FF99SB force field, post-processed with the MBAR method, and IT-TI with the GROMOS96 force field.

**Figure 4:**
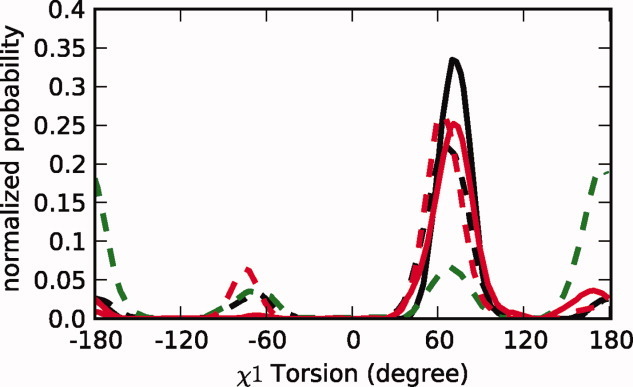
Y347 torsion changes during free energy calculations for the AMBER FF99SB ion-bound calculation (black, solid lines), AMBER FF99SB ion-free calculation with Y347 “in” starting structure (black, dashed), and with Y347 “out” starting structure (green, dashed); Also, GROMOS96 ion-bound simulations (red, solid lines) and GROMOS96 ion-free simulations (red, dashed).

For comparison to the ion-bound FEP results (Table III) and to investigate the contribution of Y347 to the binding free energy, two sets of free energy calculations for the ion-free tetramer were carried out. The first calculation was initialized from a calcium-free structure selected after 5 ns of equilibration. During this equilibration period, Y347 had not yet flipped out of the pocket for three of the four monomers (see Supporting Information Fig. 2) and monitoring of the torsion throughout this calculation indicated 17% occurrence of the “out” Y347 state, compared to 6% in the calcium-bound simulation (black solid vs. black dashed in Fig. .4). Here, the free energy difference for the ion-bound and ion-free complexes is very small (Table III), which suggests that the AMBER FF99SB force field confers stability to other binding site residues and preserves an effective binding pose with oseltamivir. However, when cluster two (Table II) of the AMBER FF99SB ion-free MD simulations was selected as starting structure for each monomer in a second tetramer calculation, simulation time spent in the Y347 “out” conformation increased to 76% (green dashed line, Fig. .4). This increased destabilization of the active site framework gives more impact on the free energy of binding, with a +2.7 kcal/mol reduced favorability in ΔΔ*G*_bind_. The change in results underscores both the influence of starting structure on sampling in free energy calculations, as well as the difficulty in achieving complete sampling using traditional MD methods.

**Table III:** Binding Free Energies (kcal/mol)

	Ion-bond			Ion-free				
Δ*G*_bind_	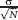 a	Δ*G*_bind_	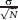 a
AMBER FF99SB with Y347 in	−14.5	1.5	−14.3	0.7
AMBER FF99SB with Y347 out			−118	1.3
GROMOS96	−15.0	2.1	−10.4	1.2
Experiment^b^	−13.7	0.2	n/a	

a *N* = 4 for AMBER calculations and *N* = 6 for GROMOS calculations (see Methods).

b Derived from *K*_*i*_ value by Kati *et al*.^41^

The TI results are an average of six monomer calculations, initiated by structures that capture diverse conformations of Loop 150, as well as varied conformations of Y347. For the ion-free complexes, three of the starting structures include a Y347 “out” conformation, and the population of this conformation increases from 13% to 20% in the ion-free calculations (Fig. .4). This population shift is not as significant as seen for the GROMOS96 MD (Fig. .2), due to the shorter timescale of the sampling in each TI λ window and fewer independent trajectories (6 for IT-TI, 10 for the MD). Still, the calcium-free Δ*G*_bind_ is +4.6 kcal/mol less favorable than the ion-bound free energy value (Table III). Structural evidence for the larger impact of the calcium presence on the GROMOS96 free energy compared to the AMBER FF99SB results can be found in the more pronounced disruptions of the active site framework, a more significantly perturbed binding pose, and overall more flexibility observed in the GROMOS96 MD. However, the discrepancy between the results for the two force fields is within the reported, propagated error.

The free energy results, listed in Table III, are a close match to each other and reflect a very high, nanomolar binding affinity. From a survey of neuraminidase activity assays in the literature42–53 the average IC_50_ value for oseltamivir binding to N1 is ∼0.2 n*M*. In Table III, we compare the calculated results with a free energy value derived from the N1-oseltamivir *K*_*i*_ in a kinetic study by Kati *et al*.^41^ Both free energy results are close to this value, within the reported error. Because all binding assays in the literature include calcium salts in the activity buffer, we lack a direct experimental comparison for the ion-free results, but the reduced favorability, seen in the GROMOS free energy results and the AMBER results for predominantly “out” Y347 conformations (Table III), corresponds to an increase in *K*_*i*_ by ∼2 orders of magnitude (from Δ*G*_bind_ = +*RT*ln*K*_*i*_).

The free energy calculations, considered with the structural changes observed in the MD simulations, implicate calcium as an important factor in maintaining an effective N1 active site framework, and, in particular, through stabilization of Y347. The “in” conformation of this residue seems to “clamp” the ligand into a favorable binding pose, which includes consistent interactions with R292 and the rest of the R triad, as well as orientation of oseltamivir such that its branched aliphatic chain is directed to a nearby hydrophobic subpocket. The sensitivity of neuraminidase binding affinity to changes in this region of the active site is also evidenced by the oseltamivir-resistant mutations of structurally neighboring residues: R292K (N2), N294S (N1), and H274Y (N1).^4^ Y347 has been suggested^1^ to prevent oseltamivir resistance to the R292K mutation in most N1 strains by providing extra stability to the binding pose, as is observed in our simulations and supported by free energy calculations. However, Y347 is mutated to an asparagine in the N1 protein of both the 1918 epidemic influenza strain and one of examined 2009 “swine flu” strains,^16^ which could allow susceptibility for resistance mutations.

## CONCLUSION

Consistent structural changes observed in simulations with two different force fields give reinforced evidence for the structural role of calcium in stabilization of the N1 active site. Free energy results calculated using different methods and force fields agree closely with each other and with experiments to indicate a contribution of 3–5 kcal/mol to the binding free energy, or two orders of magnitude in *K*_*i*_. The methods in this study also used multiple, independent simulations, initiated from diverse starting structures, in an effort to enhance sampling of the MD and obtain more accurate and reliable free energy results. Even on timescales as short as 1 ns (GROMOS96 TI), absence of the calcium causes disruption of the active site, particularly in the flipping “out” of Y347, and perturbation of the ligand binding pose. Integrity of the active site is essential for structural analysis and modeling of the N1-drug interface, often used in rational drug design. We emphasize the importance of the calcium for maintenance of this integrity and urge that it should not be excluded in N1 active site studies, particularly for structures used in docking and other computational methods. For the challenges of emerging resistance mutations in N1, which have so far occurred in the region of the active site near the bound calcium, noting the importance of framework residues in this area could also guide design of new, robust inhibitors.

## Footnotes

* http://www.cdc.gov/flu/avian/gen-info/flu-viruses.htm.

† http://simtk.org/home/pymbar
