# Supplementary material for: Impact of calcium on N1 influenza neuraminidase dynamics and binding free energy
Source: Proteins. 2010 Mar 5;78(11):2523–32. doi: 10.1002/prot.22761 (PMC2902668; doi:10.1002/prot.22761)
Supplement: Supplementary file 1 [file prot0078-2523-SD1.pdf]

Impact of calcium on N1 influenza neuraminidase dynamics and binding free energy

Morgan Lawrenz,* Jeff Wereszczynski, Rommie Amaro, Ross C. Walker, Adrian Roitberg, and J. Andrew McCammon

E-mail: mlawrenz@ucsd.edu

Supporting Information

Tables and Figures

Table 1: % Change in N1-oseltamivir Hydrogen Bonds in Ion-free simulations

	R118[a]	R292[a]	R371[a]	Y347[a]	E119[b]	E227[b]	E227[c]	E277[b]	E277[c]
AMBER FF99SB	+6	-13	-13	-14	-30	n/a	n/a	n/a	n/a
GROMOS96	-8	-40	-21	-27	-18	-49	-16	-35	-3

	Y406[a]	D151[b]	R152[c]	R144[c]
AMBER FF99SB	+3	-67	-48	n/a
GROMOS96	-4	+37	+33	-12

[a] hydrogen bond to ligand carboxyl group
[b] hydrogen bond to ligand ammonium group
[c] hydrogen bond to ligand acetamide group

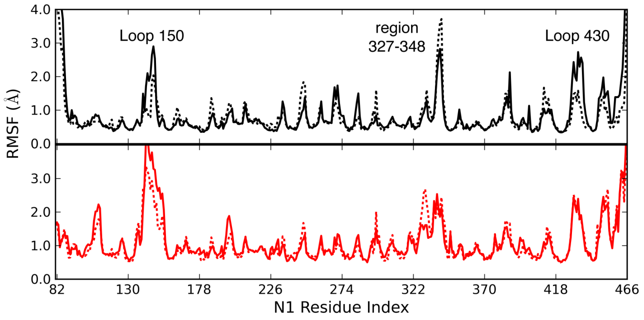

Figure 1: RMSF per residue Cα for AMBER FF99SB (black) and GROMOS96 (red) ion-bound (solid lines) and ion-free (dashed lines) simulations. Regions with high fluctuation are labeled.

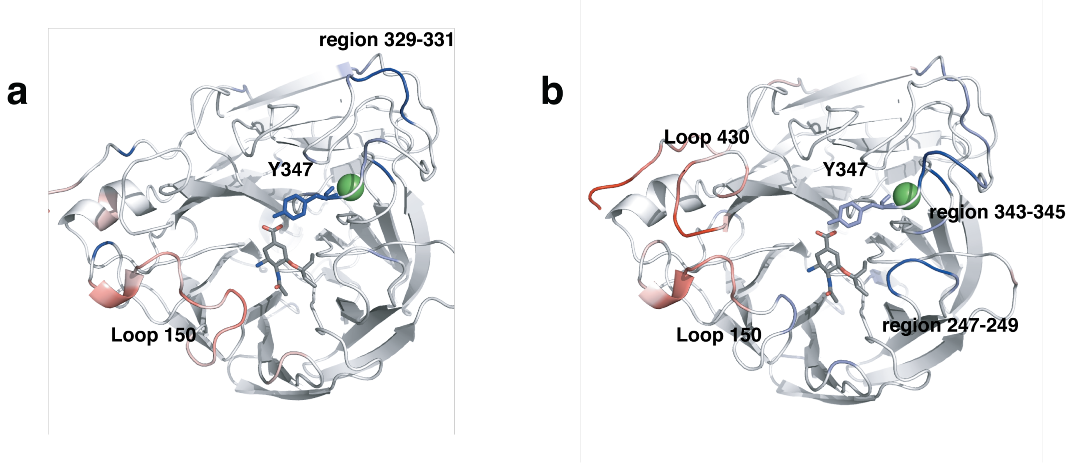

Figure 2: Changes in RMSF per residue Cα represented in B factor coloring for (a) GROMOS96 and (b) AMBER FF99SB simulations. Red indicates reduced fluctuations in the ion-free simulations, and blue indicates increased fluctuations in the ion-free simulations. Only changes in fluctuation of > 0.5 Å are colored, with the color shade deepened in intervals of 0.25 Å in the range -1.5 (red) to +1.5 (blue) Å.

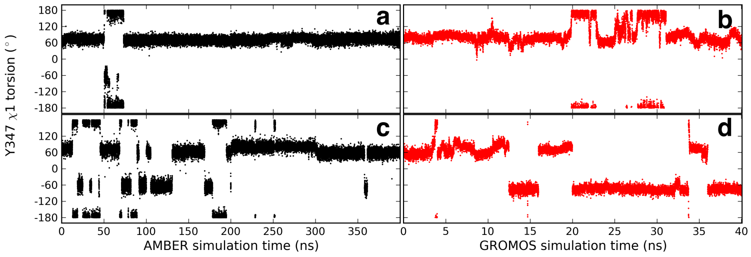

Figure 3: Time Series of Y347 $\chi 1$ torsion. Torsion is monitored over four, 100-ns concatenated monomer AMBER FF99SB simulations (black) and ten, 4-ns concatenated GROMOS96 monomer simulations (red) of calcium-bound (a,b) and calcium free (c,d) N1-oseltamivir complexes.

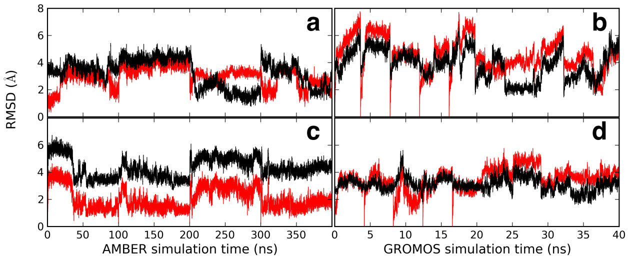

Figure 4: RMSD of Loop 150 (residues 147-152) backbone atoms from open (PDB ID:2HU0, red) and closed (PDB ID:2HU4, black) reference crystal structures, during calcium-bound (a,b) and calcium-free (c,d) GROMOS96 and AMBER FF99SB simulations.

Methods

Both calcium-bound and calcium-free N1 monomer simulations were performed using the GROMOS05 software for biomolecular simulation[1] and the GROMOS96 force field (45A3 parameter set).[2] Parameters for oseltamivir were derived from existing building blocks[3] (Table S2). Amino acid charges were defined to reproduce an apparent pH 7. The systems were solvated in boxes of SPC water molecules, with a 12 Å barrier to the periodic boundary of the cube, and neutralized with sodium ions. For the ion-bound simulations, the calcium was parametrized in the classical force field, with all ion parameters taken from those developed by Åqvist.[4]

After 2,000 steps of steepest descent energy minimization, the system was then brought to the reference temperature of 300 K in six consecutive 25 ps MD periods (50 K increments), gradually

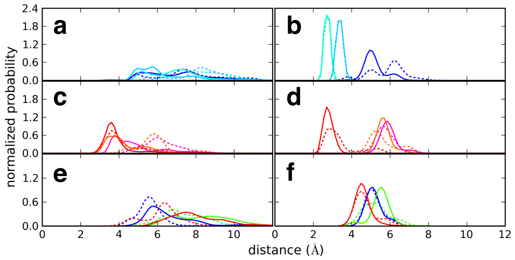

Figure 5: Distance distributions for N1-OVR reactive moieties. Distances between the centers of mass for the following moieties are monitored: a,b) R118 (cyan), R292 (blue), R371 (turquoise) guanidinium group and OVR carboxyl; c,d) E119 (red), E227 (orange), E277 (pink) and the OVR ammonium; e,f) N294 carboxamide (green), E276 carboxyl (red), and R224 (blue) guanidinium groups and the OVR aliphatic tail. Panels (a,c,e) are from GROMOS96 simulations, and right (b,d,f) are from AMBER FF99SB simulations. All solid lines denote data from ion-bound simulations, while dashed lines are from ion-free simulations.

removing a harmonic restraining potential with an initial force constant of 24 kcal$\cdot$ mol$^{-1}\cdot$Å^{-2} in increments of 4 kcal$\cdot$mol$^{-1}\cdot$Å^{-2}. Free equilibration was then carried out in the NPT ensemble (reference pressure 1 atm) by separately coupling the temperature of solute and solvent degrees of freedom to a 300 K heat bath[5] (relaxation time 0.1 ps) and by coupling the pressure (estimated based on an atomic virial) to a pressure bath[5] via isotropic coordinate scaling (relaxation time 0.5 ps). A 2 fs integration time step was used, and the SHAKE algorithm[6] was applied to constrain hydrogen containing bonds. Non-bonded interactions were re-calculated every time step in the range 0 to 8 Å and every five time steps in the range 8 to 14 Å, with truncation of these interactions at 14 Å. A reaction-field correction was applied to account for the neglected interactions beyond 14 Å,[7] using a relative dielectric permittivity of 61[8] for the SPC water model. A fast grid-based pairlist-construction algorithm[9] for non-bonded interactions was employed (cell-mask edge of 4 Å), as implemented in the GROMOS05 MD++ module.[1]

Both monomer complexes of N1-oseltamivir, with and without the bound calcium ion, were simulated in ten independent trajectories, each 4 ns, for a total of 40 ns of simulation for each complex. Comprising the ten simulations were five simulations generated from the chain B monomer in the holo Loop 150 “open” crystal structure (PDBID: 2HU0) and five simulations started from

the chain A monomer in the holo Loop 150 "closed" crystal structure (PDBID: 2HU4).[10] As the calcium density was not present in these crystal structures, overlap with the apo N1 structure 2HTY aided in positioning of the ion in the protein. After minimization, these structures were each initialized with random velocities assigned from a Maxwell-Boltzmann distribution at 5 K to generate the independent trajectories.

Calcium-bound 100 ns N1 tetramer simulations were performed with the PMEMD module in AMBER 10[11] and the AMBER FF99SB force field.[12] Atomic coordinates were taken from the holo, open Loop 150 crystal structure (2HU0), with the calcium inserted from overlap with the apo 2HTY structure and parameterized in the classical force field. Protonation states for histidines and other titratable groups were determined at pH 6.5 by the PDB2PQR[13] web server and manually verified. The tetrameric 2HU0 crystal structure has a single oseltamivir molecule bound in the active site of chain B. In order to introduce the oseltamivir within each of the other chains, chain B was aligned to chain A, C, and D using VMD[14] and the resulting transformation matrix was applied to the oseltamivir molecule. Oseltamivir was parameterized according to quantum chemical calculations, which included performing a geometry optimization with Gaussian03[15] at the Hartree-Fock/6-31G* level. The resulting atomic partial charges were then determined according to the RESP method,[16] and the atom types were assigned by the Antechamber module of AMBERTools 1.2. The GAFF[17] force field within AMBER was employed to generate the bond, angle, and dihedral parameters. As no water molecules were reported in the 2HU0 structure, we structurally aligned the 2HTY and 2HU0 systems and kept all crystallographic water molecules that did not clash with oseltamivir in the binding pocket. The system was built using the AMBER9 program Leap and the Amber99SB force field. Each monomer chain contained 8 disulfide bonds, which were properly enforced using the CYX notation in AMBER. A box of TIP3P[18] waters was added to solvate each system, resulting in a rectangular box of dimensions 124 x 127 x 77 $Å^3$. The system was neutralized with the addition of sodium counter ions and a 150 mM NaCl salt bath was introduced.

The constructed N1 ion-bound tetramer complex was first subjected to 2000 steps of steep-

est descent, followed by 5000 steps of conjugate gradient minimization using 5 kcal·mol^{-1}· Å^{-2} harmonic restraints on all non-hydrogen protein atoms. Then, 5000 steps of conjugate gradient minimization with just the backbone atoms restrained cleaned up the initial hydrogenated complex. A further 25,000 conjugate gradient minimization steps were then performed on the entire complex, without restraints, in order to alleviate any steric clashes prior to performing molecular dynamics. Following minimization, the system was linearly heated to 310 K in the NVT ensemble using a Langevin thermostat, with a collision frequency of 1.0 ps^{-1}, and harmonic restraints of 4 kcal·mol^{-1}· Å^{-2} on the backbone atoms. Further, three 250 ps periods were run in the NPT ensemble with the restraint force constant being reduced by 1 kcal·mol^{-1}· Å^{-2} each time. A final 250 ps of NPT dynamics was run without restraints. Production runs were then made for 100 ns duration in the NVT ensemble; temperature was controlled with a Langevin thermostat (1.0 ps^{-1} collision frequency), and pressure was controlled using a Berendsen Barostat[5] with a coupling constant of 1 ps and a target pressure of 1 atm. The time step used was 2 fs and all hydrogen atoms were constrained using the SHAKE algorithm.[6] Long range electrostatics were included using the Particle Mesh Ewald algorithm[19] with a 4th order B-spline interpolation and a grid spacing of <1.0 Å and a direct space cutoff of 8 Å. The trajectories for each monomer of the tetramer were extracted and concatenated to approximate 400 ns of monomer N1 sampling.

The ion-free tetramer system was simulated with atomic coordinates and parameters identical to that described above for the AMBER10 simulations, except for calcium presence, and using the AMBER ff99SB force field and the Desmond MD engine developed by D. E. Shaw Research.[20] The Maestro modeling suite was utilized for system construction in an 128 x 130 x 80 Å^3 orthorhombic box, for a minimum distance of 12 Å between protein heavy atoms and box edges. Sodium and chloride ions were added to neutralize the system charge and create an approximately 150 mM NaCl solution, as in the AMBER10 simulation. Following 10,000 steps of steepest-decent minimization, the systems were equilibrated with restraints of 10 kcal· mol^{-1}·Å^{-2} for 50 ps followed by 200 ps in which the restraints were continuously and slowly removed; then, unrestrained molecular dynamics were performed for 99.75 ns. Numerical integration was performed with a 2 fs

timestep while utilizing the M-SHAKE algorithm for constraining hydrogen containing bonds.[21] Short range interactions were truncated at 12 Å, while long range electrostatics were calculated via Particle-Mesh Ewald[19] with a 6th order B-spline used for interpolation and grid spacing <1 Å in each dimension. Constant pressure and temperature were maintained with Berendsen temperature and pressure control[5] with a reference temperature and pressure of 300 K and 1 atm.

References

(1) Christen, M.; Hünenberger, P. H.; Bakowies, D.; Baron, R.; Bürgi, R.; Geerke, D. P.; Heinz, T. N.; Kastenholz, M. A.; Kräutler, V.; Oostenbrink, C.; Peter, C.; Trzesniak, D.; van Gunsteren, W. F. *J. Comput. Chem.* **2005**, *26*, 1719–51.

(2) Schuler, L. D.; Daura, X.; van Gunsteren, W. F. *Journal of Computational Chemistry* **2001**, *22*, 1205–1218.

(3) van Gunsteren, W. F.; Billeter, S. R.; Eising, A. A.; H´unenberger, P. H.; Kruger, P.; Mark, A. E.; Scott, W. R. P.; Tironi, I. G. *Biomolecular Simulation: The GROMOS96 Manual and User Guide*; University of California, San Francisco: Zurich, Groningen, 1996.

(4) Aaqvist, J. *The Journal of Physical Chemistry* **1990**, *94*, 8021–8024, doi: 10.1021/j100384a009.

(5) Berendsen, H. J. C.; Postma, J. P. M.; van Gunsteren, W. F.; DiNola, A.; Haak, J. R. *The Journal of Chemical Physics* **1984**, *81*, 3684–3690.

(6) Ryckaert, J. P.; Ciccotti, G.; Berendsen, H. J. C. *Journal of Computational Physics* **1977**, *23*, 327–341.

(7) Tironi, I. G.; Sperb, R.; Smith, P. E.; van Gunsteren, W. F. *Journal of Chemical Physics* **1995**, *102*, 5451–5459.

(8) Heinz, T. N.; Gunsteren, W. F.; Hünenberger, P. H. *Journal of Chemical Physics* **2001**, *115*, 1125–1136.

(9) Heinz, T. N.; Hünenberger, P. H. *Journal of Computational Chemistry* **2004**, *25*, 1474–1486.

(10) Russell, R. J.; Haire, L. F.; Stevens, D. J.; Collins, P. J.; Lin, Y. P.; Blackburn, G. M.; Hay, A. J.; Gamblin, S. J.; Skehel, J. J. *Nature* **2006**, *443*, 45–49.

(11) Case, D. A. et al. *AMBER 10*; University of California, San Francisco, 2008.

(12) Hornak, V.; Abel, R.; Okur, A.; Strockbine, B.; Roitberg, A.; Simmerling, C. *Proteins* **2006**, *65*, 712–25.

(13) Dolinsky, T. J.; Nielsen, J. E.; McCammon, J. A.; Baker, N. A. *Nucleic Acids Res* **2004**, *32*, W665–7.

(14) Humphrey, W.; Dalke, A.; Schulten, K. *Journal of molecular graphics* **1996**, *14*, 33–8, 27–8.

(15) Frisch, M. J. et al. *Gaussian 03*; 2004, Gaussian, Inc., Wallingford, CT.

(16) Bayly, C. I.; Cieplak, P.; Cornell, W.; Kollman, P. A. *The Journal of Physical Chemistry* **1993**, 10269–10280.

(17) Wang, J.; Wolf, R. M.; Caldwell, J. W.; Case, P. A. K. D. A. *Journal of Computational Chemistry* **2004**, *25*, 1157–1174.

(18) Jorgensen, W. L.; Chandrasekhar, J.; Madura, J. D.; Impey, R. W.; Klein, M. L. *The Journal of Chemical Physics* **1983**, *79*, 926–935.

(19) Darden, T.; York, D.; Pedersen, L. *The Journal of Chemical Physics* **1993**, *98*, 10089–10092.

(20) Bowers, K. J.; Chow, E.; Xu, H.; Dror, R. O.; Eastwood, M. P.; Gregersen, B. A.; Klepeis, J. L.; Kolossváry, I.; Moraes, M. A.; Sacerdoti, F. D.; Salmon, J. K.; Shan, Y.; Shaw, D. E. Scalable Algorithms for Molecular Dynamics Simulations on Commodity Clusters. *Proceedings of the ACM/IEEE Conference on Supercomputing (SC06)*, 2006.

(21) Kräutler, V.; van Gunsteren, W. F.; Hünenberger, P. H. *Journal of Computational Chemistry* **2001**, *22*, 501–508.
